# Mortalidade por quedas em idosos no Distrito Federal: características e tendência temporal no período 1996-2017

**DOI:** 10.1590/S1679-49742022000100003

**Published:** 2022-03-11

**Authors:** Fabiana Medeiros de Almeida Silva, Marisete Peralta Safons

**Affiliations:** 1 Universidade de Brasília, Programa de Pós-Graduação em Educação Física, Brasília, DF, Brasil Universidade de Brasília Universidade de Brasília Programa de Pós-Graduação em Educação Física Brasília DF Brazil

**Keywords:** Acidentes por Quedas, Idoso, Registros de Mortalidade, Epidemiologia Descritiva, Accidentes por Caídas, Anciano, Registros de Mortalidad, Epidemiología Descriptiva, Accidental Falls, Aged, Mortality Registries, Epidemiology, Descriptive

## Abstract

**Objetivo:**

Descrever e analisar a tendência temporal dos óbitos por quedas em idosos no Distrito Federal, Brasil, no período de 1996 a 2017.

**Métodos:**

Estudo descritivo, a partir de dados sobre óbitos por quedas do Sistema de Informações sobre Mortalidade, da base de dados do Departamento de Informática do Sistema Único de Saúde do Brasil. Foram investigadas variáveis demográficas, socioeconômicas, tipo de queda e local de ocorrência do óbito. Realizou-se regressão linear segmentada para analisar a variação percentual anual (VPA), adotando-se p≤0,05.

**Resultados:**

Foram analisados os dados de 2.828 óbitos por quedas em idosos (mulheres, 54,2%; homens, 45,8%). Observou-se aumento do número de óbitos por quedas entre idosos com 80 anos ou mais (VPA=3,0; p<0,001).

**Conclusão:**

Houve tendência crescente de mortalidade por quedas em idosos ≥80 anos. São necessárias estratégias para redução dos óbitos por quedas, principalmente entre os idosos com idade mais avançada.

## Resumen

**Objetivo:**

Describir y analizar la tendencia temporal de muertes por caídas en ancianos en el Distrito Federal, Brasil, entre 1996 y 2017.

**Métodos:**

Estudio descriptivo basado en datos de muertes por caídas del Sistema de Información de Mortalidad, de la Base de datos del Departamento de informática del Sistema Único de Salud de Brasil. Se investigaron datos demográficos, socioeconómicos, tipo de caída y lugar de muerte. Se realizó una regresión lineal segmentada para analizar la variación porcentual anual (APC), adoptando p≤0.05.

**Resultados:**

Se analizaron los datos de 2.828 muertes por caídas en ancianos (mujeres 54,2%; hombres 45,8%). Hubo un aumento de la mortalidad por caídas en los ancianos mayores de 80 años (APC 3,0; p<0,001).

**Conclusión:**

Hubo una tendencia creciente de mortalidad por caídas en los ancianos de 80 años y más. Se necesitan estrategias para reducir las muertes por caídas, especialmente entre las personas mayores.

## Abstract (en)

**Objective:**

To describe and analyze the temporal trend of deaths from falls in the elderly in the Federal District, Brazil, between 1996 and 2017.

**Methods:**

This was a descriptive study based on data on deaths from falls held on the Mortality Information System, part of the database of the Information Technology Department of the Brazilian National Health System. Demographic, socioeconomic, type of fall and place of death variables were investigated. Segmented linear regression was performed to analyze annual percent change (APC), adopting p≤0.05.

**Results:**

Data from 2,828 deaths from falls in the elderly were analyzed (54.2% women; 45.8% men). There was an increase in mortality from falls in the elderly aged 80 years and over (APC 3.0; p<0.001).

**Conclusion:**

There was an increasing trend of mortality from falls in the elderly aged 80 years and over. Strategies are needed to reduce deaths from falls, especially among older elderly people.

**Contribuições do estudo**

**Principais resultados**

Foram analisados os dados de 2.828 óbitos por quedas em idosos (sexo feminino = 54,2%; sexo masculino = 45,8%). Observou-se aumento da mortalidade por quedas nos idosos com idade a partir de 80 anos (APC=3,0; p<0,001).

**Implicações para os serviços**

A ocorrência de quedas entre idosos representa um grave problema de saúde pública, devido à frequência com que ocorre e suas consequências, que podem gerar custos sociais e econômicos para os idosos, cuidadores e serviços de saúde.

**Perspectivas**

Monitorar os óbitos por quedas entre idosos constitui uma ação relevante para medidas de prevenção e controle, assim como para a formulação de políticas públicas direcionadas, importantes para o sistema de saúde e para a sociedade.

## Introdução

A ocorrência de quedas entre idosos representa um grave problema de Saúde Pública, dada a frequência com que ocorre e suas consequências, como as fraturas de fêmur e de quadril, podendo gerar custos sociais e econômicos para os idosos, seus cuidadores e os serviços de saúde.^1^^,^^2^

Cerca de 28 a 35% dos idosos sofrem algum episódio de queda ao ano, observando-se maior proporção desses acidentes, de 32 a 42%, após os 70 anos de idade.^3^ No Brasil, em 2018, ocorreram cerca de 12 mil óbitos por quedas em pessoas acima dos 60 anos, dos quais 84% a partir de 70 anos de idade.^4^

No mesmo período do presente estudo (1996 a 2017), ocorreram 118.233 óbitos por quedas entre idosos no Brasil. O Distrito Federal representou um percentual de 2,4% dos óbitos por quedas em idosos, considerando-se todo o território brasileiro. Trata-se do segundo maior percentual da região Centro-Oeste (27%), ficando atrás apenas do estado de Goiás (42%).^5^

O monitoramento dos óbitos por quedas entre idosos constitui uma ação relevante para a tomada de medidas de prevenção e controle desses acidentes, como também para a formulação de políticas públicas direcionadas, com possíveis repercussões de importância para o sistema de saúde e a sociedade geral. 

O objetivo deste estudo foi descrever as características e analisar a tendência temporal dos óbitos por quedas em idosos, no Distrito Federal (DF), Brasil, no período de 1996 a 2017.

## Métodos

### Delineamento

Trata-se de estudo descritivo de tendência temporal dos óbitos por quedas entre idosos no DF, no período de 1996 a 2017.

O local do estudo, o DF, situa-se na região Centro-Oeste do país e ocupa uma área de 5.779,999 Km^2^, habitada por 447.957 idosos (≥60 anos).^6^

### Participantes

Foram analisados os registros de óbitos de idosos residentes no DF e registrados segundo o Capítulo XX da Classificação Estatística Internacional de Doenças e Problemas Relacionados à Saúde - Décima Revisão (CID-10), correspondente à categoria ‘Quedas’ e aos códigos de W00 a W19.^7^ De acordo com essa classificação, as quedas podem ocorrer no mesmo nível em que a vítima se encontra ou com diferença de nível, a exemplo de escadarias, escadas verticais ou de cadeiras.

### Variáveis

As variáveis investigadas foram: sexo (masculino; feminino); faixa etária (em anos: 60 a 69; 70 a 79; 80 ou mais); raça/cor da pele (branca; não branca; ignorada), escolaridade (em anos de estudo: 0; 1 a 7; 8 ou mais; ignorada); estado civil (solteiro; casado; viúvo; outro; ignorado); local de ocorrência do óbito (hospital; domicílio; via pública; outros); e categoria da CID-10 (W00 a W19).

### Fonte de dados e mensuração

A fonte de dados foi o Sistema de Informações sobre Mortalidade (SIM), da base de dados do Departamento de Informática do Sistema Único de Saúde do Brasil (Datasus), desde o primeiro até o último ano de registro (1996 a 2017).^5^ O documento padrão do SIM é a Declaração de Óbito (DO), padronizada e distribuída em três vias, pelo Ministério da Saúde, para todo o país. A DO é preenchida pelo médico ou, em sua ausência, por duas pessoas qualificadas que tenham presenciado ou verificado a morte. As DOs são coletadas pela secretaria municipal de saúde ou do estado, no estabelecimento de saúde, e seus dados são inseridos no SIM com o objetivo de subsidiar estratégias para o controle social e o desenho de políticas públicas no país.

### Métodos estatísticos

Para analisar a série temporal dos óbitos, utilizou-se, como variável dependente, o registro de óbitos devidos a quedas, por faixa etária; e como variável independente, o ano de ocorrência dos óbitos.

Foram calculadas as taxas anuais de mortalidade por quedas, brutas (número de óbitos por queda entre idosos da área de interesse no ano específico, dividido pela população por faixa etária, na mesma área e ano, multiplicado por 100 mil) e ajustadas por idade, para estimar as tendências de mortalidade. Foi utilizado o programa Joinpoint versão 4.7 no cálculo da regressão linear segmentada, para estimar a variação percentual anual (VPA) da mortalidade e identificar pontos em que há modificação da tendência.

### Aspectos éticos

Para a coleta de dados, análise e divulgação dos resultados, não foi necessário submeter o projeto do estudo à aprovação de um comitê de ética em pesquisa, por se tratar de dados de domínio público, segundo a Resolução do Conselho Nacional de Saúde (CNS) n^o^ 466, de 12 de dezembro de 2012.

## Resultados

Foram registrados 2.828 óbitos por quedas em idosos no período de estudo, sem nenhuma exclusão. Entre estes, 54,2% em mulheres e 45,8% em homens, com predominância daqueles na idade de 80 anos ou mais (58,0%), raça/cor da pele branca (54,6%), viúvos (39,6%) e com escolaridade de 1 a 7 anos (41,5%). Em relação à causa dos óbitos, observou-se maior prevalência das categorias ‘W18 - Outras quedas no mesmo nível’ (70-79 anos, 47,2%; ≥80 anos, 52,1%) e ‘W19 - Queda sem especificação’ (60-69 anos, 35,4%) (Tabela 1).

**Tabela 1:** Características demográficas e socioeconômicas da amostra e causas de óbitos por quedas entre idosos (n=2.828), Distrito Federal, 1996-2017

Variáveis	Faixa etária (anos) n (%)		
	60-69	70-79	≥80
**Sexo**			
Masculino	326 (71,0)	377 (52,0)	591 (36,0)
Feminino	132 (29,0)	350 (48,0)	1052 (64,0)
**Raça/cor da pele**			
Branca	171 (37,0)	362 (50,0)	1011 (61,5)
Não Branca	268 (59,0)	336 (46,0)	586 (35,7)
Ignorada	19 (4,0)	29 (4,0)	46 (2,8)
**Escolaridade (anos)**			
0	89 (19,0)	184 (25,0)	525 (32,0)
01-07	195 (43,0)	325 (45,0)	653 (40,0)
≥8	110 (24,0)	213 (29,0)	268 (16,0)
Ignorada	64 (14,0)	5 (1,0)	197 (12,0)
**Estado civil**			
Solteiro	112 (24,0)	149 (20,5)	339 (21,0)
Casado	214 (47,0)	294 (40,4)	372 (23,0)
Viúvo	64 (14,0)	212 (29,2)	844 (51,0)
Outro	62 (14,0)	66 (9,1)	69 (4,0)
Ignorado	6 (1,0)	6 (0,8)	20 (1,0)
**Causa do óbito**			
W01: Queda no mesmo nível por tropeção, escorregão ou passos falsos	11 (2,4)	15 (2,1)	45 (2,7)
W03: Outras quedas no mesmo nível por colisão com ou empurrão por outra pessoa	-	-	2 (0,1)
W05: Queda envolvendo uma cadeira de rodas	2 (0,4)	2 (0,2)	13 (0,8)
W06: Queda de um leito	12 (2,6)	21 (2,9)	70 (4,3)
W07: Queda de uma cadeira	2 (0,4)	4 (0,6)	10 (0,6)
W08: Queda de outro tipo de mobília	2 (0,4)	1 (0,1)	9 (0,6)
W10: Queda em ou de escadas ou degraus	10 (2,2)	19 (2,6)	18 (1,1)
W11: Queda em ou de escadas de mão	14 (3,1)	4 (0,6)	2 (0,1)
W12: Queda em ou de um andaime	3 (0,7)	4 (0,6)	-
W13: Queda de ou para fora de edifícios ou outras estruturas	67 (14,7)	19 (2,6)	10 (0,6)
W14: Queda de árvore	12 (2,6)	5 (0,7)	2 (0,1)
W15: Queda de penhasco	-	2 (0,2)	-
W16: Mergulho-pulo em água causando outros traumas no afogamento submerso	1 (0,2)	-	-
W17: Outras quedas de um nível a outro	7 (1,5)	3 (0,4)	2 (0,1)
W18: Outras quedas no mesmo nível	153 (33,4)	343 (47,2)	856 (52,1)
W19: Queda sem especificação	162 (35,4)	285 (39,2)	604 (36,8)

A análise dos óbitos por quedas entre idosos, segundo ano e local de ocorrência, revelou que o principal local de ocorrência do evento foi o hospital (94,8%), seguido pelo domicílio (4,1%), em todos os anos da série (Tabela 2).

**Tabela 2:** Óbitos por quedas entre idosos (n=2.828) por ano, segundo local de ocorrência, Distrito Federal, 1996-2017

Ano	Hospital	Domicílio	Via pública	Outros
	n (%)	n (%)	n (%)	n (%)
**1996**	42 (91,3)	4 (8,7)		-
**1997**	47 (97,9)	1 (2,1)	-	-
**1998**	37 (90,2)	4 (9,8)	-	-
**1999**	38 (95,0)	2 (5,0)	-	-
**2000**	62 (95,4)	2 (3,1)	-	1 (1,5)
**2001**	74 (100,0)	-	-	-
**2002**	73 (88,0)	8 (9,6)	1 (1,2)	1 (1,2)
**2003**	62 (87,3)	7 (9,9)	-	2 (2,8)
**2004**	113 (96,6)	3 (2,6)	1 (0,9)	-
**2005**	122 (96,1)	4 (3,1)	-	1 (0,8)
**2006**	123 (95,3)	5 (3,9)	1 (0,8)	-
**2007**	99 (95,2)	5 (4,8)	-	-
**2008**	145 (98,0)	3 (2,0)	-	-
**2009**	162 (97,6)	3 (1,8)	1 (0,6)	-
**2010**	182 (93,3)	11 (5,6)	2 (1,0)	-
**2011**	151 (94,4)	8 (5,0)	1 (0,6)	-
**2012**	155 (92,3)	10 (6,0)	1 (0,6)	2 (1,2)
**2013**	175 (93,6)	11 (5,9)	-	1 (0,5)
**2014**	193 (95,1)	7 (3,4)	1 (0,5)	2 (1,0)
**2015**	175 (96,7)	4 (2,2)	-	2 (1,1)
**2016**	224 (95,3)	9 (3,8)	-	2 (0,9)
**2017**	228 (95,0)	5 (2,1)	1 (0,4)	6 (2,5)
**Total**	**2.682 (94,8)**	**116 (4,1)**	**10 (0,4)**	**20 (0,7)**

A análise de tendência de óbitos por quedas, segundo as faixas etárias definidas (60-69, 70-79 e ≥80 anos), demonstrou pontos de inflexão positiva na curva de tendência estatisticamente significantes apenas na população a partir de 80 anos de idade (VPA=3,0 - IC_95%_ 1,2;4,9 - p<0,001). Observou-se diminuição (60 a 69 anos) e aumento (70 a 79 anos) na tendência de óbitos por quedas, embora esses resultados não fossem estatisticamente significativos (Tabela 3).

**Tabela 3:** Variação percentual anual (VPA) das taxas de óbitos por quedas entre idosos (n=2.828) por grupo etário e intervalo de confiança de 95% (IC_95%_), Distrito Federal, 1996-2017

Faixa etária (anos)	VPA (IC_95%_)	Valor-p^a^
**60-69**	-0,7 (-3,0;1,7)	0,690
**70-79**	0,5 (-1,1;2,1)	0,510
**≥80**	3,0 (1,2;4,9)	<0,001

a) Teste de regressão *joinpoint*.

## Discussão

No DF, entre os anos de 1996 e 2017, os óbitos por quedas aumentaram com o envelhecimento, sendo mais frequentes nos idosos com 80 anos ou mais idade, comparados aos de 60 a 69 anos. Houve maior proporção dos óbitos por quedas entre mulheres, nas idades mais avançadas, entre os(as) viúvos(as) e aqueles(as) com baixa escolaridade. O ambiente hospitalar foi o local de ocorrência mais frequente dos casos notificados de óbitos por quedas, sendo ‘Outras quedas do mesmo nível’ o tipo de queda mais frequente. No período estudado (1996-2017), houve tendência de aumento dos óbitos por quedas entre os idosos com 80 anos ou mais.

A presente pesquisa baseia-se em dados secundários e, portanto, é passível de erros decorrentes de digitação e registro. Entretanto, por se tratar de dados nacionais oficiais, de preenchimento obrigatório em todos os serviços de saúde, acredita-se que as informações são confiáveis e permitiram o alcance dos objetivos propostos.

Consistentemente com relatos da literatura nacional e internacional, a maioria dos óbitos correspondeu a mulheres, sendo as possíveis causas (i) a menor quantidade de massa magra e de força muscular, em relação aos idosos homens, e (ii) a maior perda de massa óssea, devido à redução do estrógeno, fatores esses associados à fragilidade e risco de fratura.^8^^-^^10^

A maior ocorrência dos óbitos em idosos com idade mais avançada pode estar relacionada às alterações fisiológicas resultantes do avanço da idade, caracterizadas pela diminuição da massa óssea e muscular e aumento do tecido adiposo, processos que podem comprometer o funcionamento do sistema musculoesquelético,^11^ ao uso de medicamentos psicotrópicos, como antipsicóticos e antidepressivos,^12^ e à polifarmácia (uso de cinco ou mais medicamentos).^13^

Os óbitos por quedas ocorreram principalmente entre idosos viúvos, corroborando dados da literatura. Estudos nacionais de base populacional e longitudinal, sobre fatores associados às quedas, apontam que viver com companheiro(a) pode resultar em cuidado mútuo e menor ocorrência desses eventos.^14^^,^^15^ Com base em 17 estudos longitudinais e transversais, cujas amostras variaram de 200 a 43.367 idosos, publicados entre 2003 e 2019, uma revisão sistemática demonstrou que solidão, isolamento social e morar sozinho foram fatores significativamente associados às quedas em idosos.^16^

A ocorrência de óbitos por quedas em idosos foi mais elevada entre os alfabetizados, porém com baixa escolaridade (1 a 7 anos), e que, na maioria das vezes, vivem com menor renda e condições mínimas de vida e saúde. Este resultado está de acordo com outros estudos, os quais também identificaram o baixo nível de escolaridade como fator de risco para quedas, enquanto o alto nível de escolaridade como fator protetor para limitação da mobilidade entre idosos.^17^^-^^19^

Os óbitos por quedas no ambiente hospitalar representaram, aproximadamente, 95% dos casos. Entretanto, essa informação não é suficiente no contexto da prevenção de quedas, pois é importante conhecer o local de ocorrência para melhor direcionamento das estratégias de modificação ambiental. Apesar de esse dado não ser fornecido pelo SIM, estudos nacionais e internacionais, sobre prevalência e determinantes das quedas em idosos, demonstram que as quedas acidentais ocorrem, na maioria das vezes, dentro da própria residência do idoso ou em seus arredores, durante a realização das atividades cotidianas.^18^^,^^20^^-^^22^

Outras quedas do mesmo nível foi o tipo de queda mais frequente, cerca de metade dos óbitos por quedas, coincidindo com dados encontrados na literatura.^20^^,^^23^ Esse tipo de queda pode ocorrer devido a fatores intrínsecos (história prévia de quedas, avanço da idade, uso de medicamentos, presença de doenças metabólicas, neurológicas ou osteoarticulares, deficiência visual, dependência funcional) e extrínsecos (iluminação inadequada, superfícies escorregadias, obstáculos, tapetes, degraus, ausência de corrimãos).^24^ Foi observado elevado número de óbitos por quedas sem as devidas especificações, possivelmente resultante de falhas na qualidade das informações registradas nas declarações de óbito - DOs. 

Observou-se aumento na tendência da mortalidade por quedas nos idosos a partir de 80 anos, resultado semelhante ao de outros estudos.^25^^-^^28^ Um estudo ecológico nacional, realizado entre 1996 e 2012, observou aumento de 200% na taxa de mortalidade de idosos em decorrência de quedas, nas capitais brasileiras;^25^ outro estudo, este realizado com dados do Sistema de Informações Hospitalares do Sistema Único de Saúde (1998 a 2015), observou tendência crescente das taxas de internação, mortalidade e letalidade por quedas em idosos (4,5%).^26^

Em conclusão, observou-se tendência crescente na mortalidade por quedas em idosos com 80 anos ou mais, no Distrito Federal, entre 1996 e 2017. Esse aumento pode ser reflexo de mudanças nos perfis demográfico, socioeconômico e comportamental, associadas ao envelhecimento populacional. São necessárias estratégias para redução dos óbitos por quedas, principalmente entre os idosos com idade mais avançada, como a utilização da Caderneta de Saúde da Pessoa Idosa, um documento do Sistema Único de Saúde - SUS - que possibilita a identificação do risco de quedas e oferece orientações para o autocuidado.
