# Supplementary material for: Mortality from falls in the elderly in the Federal District, Brazil: characteristics and time trend, 1996-2017
Source: Epidemiol Serv Saude. 2022 Mar 11;31(1):e2021681. [Article in Portuguese] doi: 10.1590/S1679-49742022000100003 (PMC11446288; doi:10.1590/S1679-49742022000100003)
Supplement: Translation

# Translation: Mortality from falls in the elderly in the Federal District, Brazil: characteristics and time trend, 1996-2017

**Authors:** Fabiana Medeiros de Almeida Silva, Marisete Peralta Safons

**Affiliations:** 1 Universidade de Brasília, Programa de Pós-Graduação em Educação Física, Brasília, DF, Brazil Universidade de Brasília Universidade de Brasília Programa de Pós-Graduação em Educação Física Brasília DF Brazil

**Keywords:** Accidental Falls, Aged, Mortality Registries, Epidemiology, Descriptive

## Abstract

**Objective:**

To describe and analyze the temporal trend of deaths from falls in the elderly in the Federal District, Brazil, between 1996 and 2017.

**Methods:**

This was a descriptive study based on data on deaths from falls held on the Mortality Information System, part of the database of the Information Technology Department of the Brazilian National Health System. Demographic, socioeconomic, type of fall and place of death variables were investigated. Segmented linear regression was performed to analyze annual percent change (APC), adopting p≤0.05.

**Results:**

Data from 2,828 deaths from falls in the elderly were analyzed (54.2% women; 45.8% men). There was an increase in mortality from falls in the elderly aged 80 years and over (APC 3.0; p<0.001).

**Conclusion:**

There was an increasing trend of mortality from falls in the elderly aged 80 years and over. Strategies are needed to reduce deaths from falls, especially among older elderly people.

**Research contribution**

**Main results**

Data from 2,828 deaths due to falls among the elderly (female= 54.2%; males = 45.8%). There was an increase in mortality due to falls in the elderly aged 80 years and older (PCA=3.0; p<0,001).

**Implications for services**

The occurrence of falls among the elderly represents a major public health problem, due to the frequency with which it happens and its consequences, which may generate social and economic costs for the elderly, caregivers and health services.

**Perspectives**

Monitoring deaths due to falls among the elderly is relevant for prevention and control measures, as well as the development of public policies, important for the health system and society.

## Introduction

Occurrence of falls among the elderly represents a serious Public Health problem, given the frequency with which it occurs and its consequences, such as femur and hip fractures, which can generate social and economic costs for the elderly, their caregivers and health services.^1^^,^^2^

Some 28% to 35% of elderly people suffer an episode of falling per year, with a higher proportion of these accidents, 32% to 42%, after the age of 70.^3^ In Brazil, in 2018, there were around 12,000 deaths from falls in people over 60 years of age, 84% of whom were over 70 years of age.^4^

In the same period as that covered by this study (1996-2017), there were 118,233 deaths from falls among the elderly in Brazil. The Federal District accounted for 2.4% of deaths due to falls among the elderly, considering the entire Brazilian territory. This is the second highest percentage in the country’s Midwest region (27%), coming only behind the state of Goiás (42%).^5^

Monitoring deaths from falls among the elderly is a relevant action for taking measures to prevent and control these accidents, as well as for formulating targeted public policies, with possible repercussions of importance for the health system and society in general. 

The objective of this study was to describe the characteristics and analyze the temporal trend of deaths from falls in the elderly, in the Federal District, Brazil, from 1996 to 2017.

## Methods

### Design

This is a descriptive study of the temporal trend of deaths from falls among the elderly in the Federal District from 1996 to 2017.

The study site, the Federal District, is located in the Midwest region of the Brazil and occupies an area of 5,779.999 Km^2^, inhabited by 447,957 elderly people (≥60 years old).^6^

### Participants

We analyzed the records of deaths of elderly people living in the Federal District and which were recorded according to Chapter XX of the International Statistical Classification of Diseases and Related Health Problems - Tenth Revision (ICD-10), corresponding to the ‘Falls’ category and codes W00 to W19.^7^ According to this classification, falls can occur on the same level as the victim or involve different levels, such as falling from steps, ladders or chairs.

### Variables

The variables investigated were: sex (male; female); age group (in years: 60-69; 70-79; 80 or over); race/skin color (white; non-white; unknown), schooling (in years of study: 0; 1-7; 8 or more; unknown); marital status (single; married; widowed; other; unknown); place of death (hospital; home; public thoroughfare; other); and ICD-10 category (W00 to W19).

### Data source and measurement 

The data source was the Mortality Information System, part of the database of the Information Technology Department (DATASUS) of the Brazilian National Health System, taking the records for each year studied (1996 to 2017).^5^ The standard document used to input information to the Mortality Information System is the Declaration of Death. It is a standardized document with three copies and is provided by the Ministry of Health for use throughout the entire country. The Declaration of Death is filled out by the physician or, in his/her absence, by two qualified persons who witnessed or verified the death. The Declarations of Death are collected by the municipal or state Health Department from the health facility, and the data are input to the Mortality Information System with the objective of informing strategies for public policy monitoring and design in Brazil.

### Statistical methods

In order to analyze the death time series, we used the records of deaths due to falls, by age group as the dependent variable; while the independent variable was the year in which the deaths occurred.

We calculated the annual rates of death from falls. First we calculated the crude rates (number of deaths from falls among the elderly in the area of interest in the specific year, divided by the population according to age group, in the same area and year, multiplied by 100,000). Then we calculated age-adjusted death rates in order to estimate mortality trends. We used Joinpoint version 4.7 to calculate segmented linear regression in order estimate annual percent change (APC) in mortality and identify points where there was a change in the trend.

### Ethical aspects 

In accordance with National Health Council Resolution No. 466, dated December 12, 2012, it was not necessary to submit the study project for approval by a research ethics committee with regard to data collection, analysis and publication of the results, since the study used public domain data.

## Results

There were 2,828 records of deaths from falls in the elderly during the study period, and none were excluded. Among these, 54.2% were women and 45.8% were men, with a predominance of those aged 80 years or older (58.0%), those of white race/skin color (54.6%), widowed (39.6%) and with 1 to 7 years of schooling (41.5%). Regarding cause of death, we found a higher prevalence of the following categories: ‘W18 - Other fall on same level’ (70-79 years, 47.2%; ≥80 years, 52.1%) and ‘W19 - Unspecified fall’ (60-69 years, 35.4%) (Table 1).

**Table 1:** Demographic and socioeconomic characteristics of the sample and causes of death due to falls among elderly people (n=2,828), Federal District, Brazil, 1996-2017

Variables	Age group (years) n (%)		
	60-69	70-79	≥80
**Sex**			
Male	326 (71.0)	377 (52.0)	591 (36.0)
Female	132 (29.0)	350 (48.0)	1052 (64.0)
**Race/skin color**			
White	171 (37.0)	362 (50.0)	1011 (61.5)
Non-white	268 (59.0)	336 (46.0)	586 (35.7)
Unknown	19 (4.0)	29 (4.0)	46 (2.8)
**Schooling (years)**			
0	89 (19.0)	184 (25.0)	525 (32.0)
1-7	195 (43.0)	325 (45.0)	653 (40.0)
≥8	110 (24.0)	213 (29.0)	268 (16.0)
Unknown	64 (14.0)	5 (1.0)	197 (12.0)
**Marital status**			
Single	112 (24.0)	149 (20.5)	339 (21.0)
Married	214 (47.0)	294 (40.4)	372 (23.0)
Widowed	64 (14.0)	212 (29.2)	844 (51.0)
Other	62 (14.0)	66 (9.1)	69 (4.0)
Unknown	6 (1.0)	6 (0.8)	20 (1.0)
**Cause of death**			
W01: Fall on the same level from slipping, tripping and stumbling	11 (2.4)	15 (2.1)	45 (2.7)
W03: Other fall on the same level due to collision with, or pushing by, another person	-	-	2 (0.1)
W05: Fall involving wheelchair	2 (0.4)	2 (0.2)	13 (0.8)
W06: Fall involving bed	12 (2.6)	21 (2.9)	70 (4.3)
W07: Fall involving chair	2 (0.4)	4 (0.6)	10 (0.6)
W08: Fall involving other furniture	2 (0.4)	1 (0.1)	9 (0.6)
W10: Fall on and from stairs and steps	10 (2.2)	19 (2.6)	18 (1.1)
W11: Fall on and from ladder	14 (3.1)	4 (0.6)	2 (0.1)
W12: Fall on and from scaffolding	3 (0.7)	4 (0.6)	-
W13: Fall from, out of or through building or structure	67 (14.7)	19 (2.6)	10 (0.6)
W14: Fall from tree	12 (2.6)	5 (0.7)	2 (0.1)
W15: Fall from cliff	-	2 (0.2)	-
W16: Diving or jumping into water causing injury other than drowning or submersion	1 (0.2)	-	-
W17: Other fall from one level to another	7 (1.5)	3 (0.4)	2 (0.1)
W18: Other fall on same level	153 (33.4)	343 (47.2)	856 (52.1)
W19: Unspecified fall	162 (35.4)	285 (39.2)	604 (36.8)

Analysis of deaths from falls among the elderly, according to year and place of occurrence, revealed that hospitals were the main place of death (94.8%), followed by deaths at home (4.1%), in all years of the time series (Table 2).

**Table 2:** Deaths due to falls among elderly people (n=2,828) by year and place of occurrence, Federal District, Brazil, 1996-2017

Year	Hospital	At Home	Public Thoroughfare	Other
	n (%)	n (%)	n (%)	n (%)
**1996**	42 (91.3)	4 (8.7)		-
**1997**	47 (97.9)	1 (2.1)	-	-
**1998**	37 (90.2)	4 (9.8)	-	-
**1999**	38 (95.0)	2 (5.0)	-	-
**2000**	62 (95.4)	2 (3.1)	-	1 (1.5)
**2001**	74 (100.0)	-	-	-
**2002**	73 (88.0)	8 (9.6)	1 (1.2)	1 (1.2)
**2003**	62 (87.3)	7 (9.9)	-	2 (2.8)
**2004**	113 (96.6)	3 (2.6)	1 (0.9)	-
**2005**	122 (96.1)	4 (3.1)	-	1 (0.8)
**2006**	123 (95.3)	5 (3.9)	1 (0.8)	-
**2007**	99 (95.2)	5 (4.8)	-	-
**2008**	145 (98.0)	3 (2.0)	-	-
**2009**	162 (97.6)	3 (1.8)	1 (0.6)	-
**2010**	182 (93.3)	11 (5.6)	2 (1.0)	-
**2011**	151 (94.4)	8 (5.0)	1 (0.6)	-
**2012**	155 (92.3)	10 (6.0)	1 (0.6)	2 (1.2)
**2013**	175 (93.6)	11 (5.9)	-	1 (0.5)
**2014**	193 (95.1)	7 (3.4)	1 (0.5)	2 (1.0)
**2015**	175 (96.7)	4 (2.2)	-	2 (1.1)
**2016**	224 (95.3)	9 (3.8)	-	2 (0.9)
**2017**	228 (95.0)	5 (2.1)	1 (0.4)	6 (2.5)
**Total**	**2,682 (94.8)**	**116 (4.1)**	**10 (0.4)**	**20 (0.7)**

Analysis of the trend of deaths from falls, according to the age groups defined (60-69, 70-79 and ≥80 years), showed statistically significant positive inflection points in the trend curve only for the population aged 80 years and older (APC=3.0 - 95%CI 1.2;4.9 - p<0.001). We found a decrease (60-69 years) and an increase (70-79 years) in the trend of deaths from falls, although these results were not statistically significant (Table 3).

**Table 3:** Annual percent change (APC) in death rates due to falls among elderly people (n=2,828) by age group and 95% confidence interval (95%CI), Federal District, Brazil, 1996-2017

Age group (years)	APC (95%CI)	p-value^a^
**60-69**	-0.7 (-3.0;1.7)	0.690
**70-79**	0.5 (-1.1;2.1)	0.510
**≥80**	3.0 (1.2;4.9)	**<0.001**

a) Joinpoint regression test.

## Discussion

Between 1996 and 2017, deaths from falls in the Federal District increased as age increased, being more frequent in the elderly aged 80 or over, compared to those who were 60 to 69 years old. There was a higher proportion of deaths due to falls among women, those who were older, widowed and had low schooling. The hospital setting was the most frequent location of reported cases of deaths from falls, with ‘Other fall on same level’ being the most frequent type of fall. In the period studied (1996-2017), there was an increasing trend of deaths from falls among the elderly aged 80 or over.

This research is based on secondary data and is, therefore, subject to typing and recording errors. However, because it is official national data, which is required to be filled out in all health services, we believe that the information is reliable and enabled the proposed objectives to be achieved.

In keeping with reports in the national and international literature, the majority of deaths related to women, the possible causes of this being (i) lower lean mass and muscle strength compared to elderly men, and (ii) greater loss of bone mass due to reduced estrogen, these being factors associated with frailty and fracture risk.^8^^-^^10^

Higher occurrence of deaths in older elderly people may be related to physiological changes resulting from advancing age, characterized by decreased bone and muscle mass and increased fat tissue, these being processes that can compromise the functioning of the musculoskeletal system,^11^ as well as being related to use of psychotropic medications, such as antipsychotics and antidepressants,^12^ and polypharmacy (use of five or more medications).^13^

Deaths from falls occurred mainly among widowed elderly people, corroborating data in the literature. National population-based and longitudinal studies on factors associated with falls indicate that living with a partner may result in mutual care and lower occurrence of these events.^14^^,^^15^ Based on 17 longitudinal and cross-sectional studies, with samples ranging from 200 to 43,367 elderly people, published between 2003 and 2019, a systematic review demonstrated that loneliness, social isolation and living alone were factors significantly associated with falls among the elderly.^16^

Occurrence of deaths from falls in the elderly was higher among those who, although they were literate, had low levels of schooling (1 to 7 years), and who, in most cases, have lower income and only basic living and health conditions. This result is in agreement with other studies, which also identified low level of schooling as a risk factor for falls, while a high level of schooling was found to be a protective factor in relation to mobility limitation among elderly people.^17^^-^^19^

Falls leading to death in the hospital environment accounted for approximately 95% of cases. However, this information is not enough in the context of fall prevention, because it is important to know where the falls occurred in order to achieve better targeting of strategies to modify environments. Although this data is not provided by the Mortality Information System, national and international studies on prevalence and determinants of falls in the elderly show that accidental falls occur, in most cases, inside the elderly person’s own home or immediate surroundings, while performing everyday activities.^18^^,^^20^^-^^22^

Other falls on the same level were the most frequent type of fall, accounting for about half of the deaths from falls. This coincides with data found in the literature.^20^^,^^23^ This type of fall can occur due to intrinsic factors (prior history of falls, advancing age, use of medications, presence of metabolic, neurological or osteoarticular diseases, visual impairment, functional dependence) and extrinsic factors (inadequate lighting, slippery surfaces, obstacles, rugs, steps, absence of handrails).^24^ We found a high number of deaths from falls that were not properly specified, possibly resulting from shortcomings in the quality of information recorded on the Declarations of Death. 

We found an increase in the trend of mortality due to falls in the elderly aged 80 and over, this being a result similar to that of other studies.^25^^-^^28^ A national ecological study, conducted between 1996 and 2012, found a 200% increase in the elderly mortality rate due to falls in Brazilian state capitals.^25^ A study conducted using data from the Brazilian National Health System Hospital Information System (1998-2015), found an increasing trend in hospitalization, mortality and case fatality rates due to falls among the elderly (4.5%).^26^

In conclusion, we found an increasing trend in mortality from falls in the elderly aged 80 and over in the Federal District between 1996 and 2017. This increase may reflect changes in demographic, socioeconomic and behavioral profiles, associated with population aging. Strategies are needed to reduce deaths from falls, especially among older adults, such as use of the Elderly Person’s Health Booklet, a record and guidance document used by the Brazilian National Health System that allows identification of risk of falls and offers guidance on self-care.
